# Effect of Orem’s Self-Care Theory Combined with Active Pain Assessment on Pain, Stress and Psychological State of Children with Nephroblastoma Surgery

**DOI:** 10.3389/fsurg.2022.904051

**Published:** 2022-05-16

**Authors:** Yuanhui Tang, Yaoyao Chen, Yanfang Li

**Affiliations:** ^1^ Department of Nursing, Hunan Children’s Hospital, Changsha, China; ^2^ Department of Urology Surgery, Hunan Children’s Hospital, Changsha, China

**Keywords:** nephroblastoma surgery, Orem’s self-care theory, active pain assessment, psychological state, stress response

## Abstract

**Background:**

With the development of medical technology and the innovation of various surgical options, the survival time of children with nephroblastoma is significantly prolonged. However, postoperative pain and stress response have been plagued by children with nephroblastoma during the postoperative treatment. At present, there is still a lack of effective care programs.

**Methods:**

We accessed our institutional database to retrospectively screen clinical data from all children with nephroblastoma who were surgically treated in our hospital between July 2020 and July 2021. Some children received routine care, while others received Orem-based self-care theory and active pain assessment.

**Results:**

According to the inclusion and exclusion criteria, 150 children with nephroblastoma who underwent surgical treatment were included in this study. On the third day after surgery, the scores of pain control effect and satisfaction degree of pain education in the study group were higher than those in the control group, and the physical and daily life influence, emotion influence, and pain experienced in the study group were lower than those in the control group. The differences were statistically significant (*p *< 0.001). There was no significant difference in C-SUPPH and ESCA scores between the two groups before nursing (*p *> 0.05). After nursing, the C-SUPPH and ESCA scores of the two groups were higher than those before nursing, and the C-SUPPH and ESCA scores of the study group were higher than those of the control group (*p *< 0.05). Before nursing, the levels of ACTH, Cor, and ANP between the two groups were not statistically significant (*p* > 0.05). The levels of ACTH, Cor, and ANP in the two groups were lower than those before nursing and 3 d and 7 d after nursing, and the index levels after 7 d of nursing were lower than those after 3 d of nursing. After nursing, the levels of ACTH, Cor, and ANP in the study group at each time point were lower than those in the control group (*p *< 0.05). There was no significant difference in SAS and SDS scores between the two groups before nursing (*p* > 0.05). After nursing, the SAS and SDS scores of both groups were lower than those before nursing, and the SAS and SDS scores of the study group were lower than those of the control group (*p *< 0.05). There was no significant difference in PSQI scores between the two groups before nursing (*p *> 0.05). After nursing, the PSQI scores of the two groups were lower than those before nursing, and the PSQI scores of the study were lower than those of the control group (*p *< 0.05). The average daily crying time, the average hospitalization time, and postoperative off-bed time in the study group were shorter than those in the control group (*p *< 0.05).

**Conclusion:**

Orem’s self-care theory combined with active pain assessment can reduce pain in children undergoing nephroblastoma surgery, improve their stress response and psychological state, and improve their sleep quality, which is conducive to postoperative recovery and worthy of promotion.

## Introduction

Nephroblastoma is an embryonal malignancy tumor with the highest incidence of abdominal tumors in children. The exact cause of the disease remains unclear (1). From the perspective of embryology, nephroblastoma develops from an abnormal proliferation of persistent postrenal blastocysts (2). Tumor tissue can break through the renal dorsal membrane and destroy the kidney skin, renal pelvis, kidney, etc., resulting in abnormal renal function. Besides, some tumor cells can invade the blood vessels of the renal hilus or even the surrounding tissues, endangering life (3). Therefore, timely surgical treatment is essential to save the lives of children. Pain is a protective mechanism of the body, which is used to restrict the postoperative activities of children undergoing surgery and avoid adverse consequences. However, the pain will not only cause a huge psychological burden but also limit other immune functions, cause different degrees of stress response, and increase the rate of postoperative lesion metastasis (4). Therefore, postoperative pain in children with nephroblastoma is the key link in postoperative nursing.

Orem’s self-care theory is a new type of nursing model. The self-care theory emphasizes self-care as the center, with the ultimate goal of enabling individuals to take up the responsibility of self-care (5). Nursing intervention based on Orem’s theory focuses on being people-oriented, which can help patients improve their self-nursing ability and maintain a good psychological state on the basis of comprehensive nursing. In addition, pain assessment is the basis of pain management. The study indicates that targeted measures based on the results of active pain assessment can effectively relieve postoperative pain in children (6). This study analyzed the influence of pain, stress, and psychological state of children with nephroblastoma surgery after surgery based on Orem’s self-care theory and active pain assessment.

## Materials and Methods

### Research Object

This study has been approved by the Ethics Committee of our hospital, and all patients have been informed and consented. All the data have been confirmed.

We accessed our institutional database to retrospectively screen clinical data from all children with nephroblastoma who were surgically treated in our hospital between July 2020 and July 2021. The inclusion criteria are as follows: (1) All children who underwent nephroblastoma surgery; (2) children aged 6–10 years;and (3) children with good communication skills and able to objectively answer questions. The exclusion criteria are as follows: (1) all patients with other malignant tumors and distant metastasis; (2) severe impairment of organ function; (3) children with cognitive impairment; (4) intraoperative and postoperative blood transfusion; and (5) children with a history of epilepsy. Clinical data deficiencies were excluded, and the cooperation with the investigator was refused in the midway. According to the different nursing methods, the participants were divided into a study group and a control group.

### Care Methods

All the children received routine pain intervention, living nursing, vital sign monitoring, condition monitoring, and incision prevention of infection after operation. Children were urged to undergo rehabilitation training as soon as possible. Medication guidance was given to children, and parents were given education on the related knowledge of active pain after surgery, explaining the causes of pain and the use of analgesic drugs. Psychological nursing was carried out to eliminate anxiety and fear of children and their families.

In the study group, Orem’s self-care theory was added to routine nursing. (1) Complete compensation system: Children lack self-nursing knowledge at the initial stage of admission. Nurses should do all kinds of nursing work to meet the normal requirements of children as much as possible. According to the actual situation, cognitive behavioral therapy, music therapy, therapeutic communication, exercise therapy, art therapy, reading therapy, virtual reality therapy, and other methods were selected to conduct psychological counseling for children. (2) Partial compensation system: At this stage, children are required to participate in activities together, and they can be required to independently take nursing of themselves, such as dressing, bathing, eating, and defecating. Encourage children to adjust their emotions in the treatment and nursing process and maintain a good psychological state. (3) Support and education system: During hospitalization, nurses should teach children self-nursing skills to improve their self-nursing ability as much as possible. Emotional and information support should be given, and medical staff should try their best to praise the children with soothing, encouraging, hinting, and praising language to improve their health awareness and treatment compliance. Children and their families should be taught self-nursing methods and precautions after discharge, emphasizing the importance of self-nursing to patients and improving the ability and enthusiasm of self-nursing.

Meanwhile, the active pain state of children was evaluated. The pain should be tracked once every 4 h. When the FAS score of the child dropped to Grade I or II and the NRS score <4, it was changed to be evaluated once every 8 h. Pain assessment should be stopped when the child’s pain disappears. The assessment tools were the Digital Pain Rating Scale (NRS) and the Chinese Version of the Four-Level Functional Activity Score (FAS) (7, 8). The NRS scale was mainly used for children to assess their own pain subjectively. The scale was composed of 0–10 numbers, with 0–3 points indicating that the pain was within the tolerance range and did not affect normal life and sleep, 4–6 points meaning that the pain is more obvious and the basic living and sleep have interfered, and 7–10 points meaning severe pain that the children cannot tolerate basically. The FAS scale is mainly used for the assessment of postoperative active pain, and it is assessed according to the degree of pain endured by children. Grade I indicates that pain could not limit the children from completing a functional activity normally. Grade II indicates that the children with mild pain restriction completed a functional activity. Grade III indicates that the pain moderately restricted the children to complete a functional activity. Grade IV indicates severe pain that limits the child’s ability to complete a functional activity, and activity is not recommended for this grade. No drug intervention was given when the FAS score was Grade I. Psychological intervention was given to divert patients’ attention to pain when the FAS score was Grade II or NRS score >4. When the FAS score was Grade III or IV or the NRS score was greater than 7, an intramuscular injection of analgesic drugs was administered.

Both groups were intervened for 3 weeks.

### Observation Index

(1) On the third postoperative day, the Houston Pain Inventory (HPOI) (9) was used to assess the pain control effects of two groups, including pain control effect, physical and daily life influence, emotional influence, pain experience, and satisfaction degree of pain education with 33 items in five dimensions. Among them, the higher the scores of pain control effect and satisfaction degree of pain education were, the more satisfied the pain control effect was. The lower the scores of body and daily life influence, emotion influence, and pain experience were, the more satisfied the pain control effect was.
(2) Before and after nursing, the self-management efficacy scale (C-SUPPH) (10) and self-nursing ability scale (ESCA) (11) were used to evaluate the level of self-efficacy and self-nursing ability of the two groups, respectively. The C-SUPPH scale consisted of 28 items in three dimensions: self-decompression, self-decision-making, and positive attitude, with each item scoring 1–5 points and a total score of 28–140 points. The higher the total score is, the higher the self-efficacy is. The ESCA scale consisted of 43 items in four dimensions: self-concept, self-nursing responsibility, self-nursing skills, and health knowledge level. Each item was scored with 0–4 points, and the total score was 0–172 points. The higher the score is, the higher the self-nursing ability is.
(3) Before nursing, after nursing for 3 days, and after nursing for 7 days, 5 mL of fasting elbow venous blood was collected, and the upper serum was centrifuged to detect the serum adrenocorticotropic hormone (ACTH), cortisol (Cor), and atrial natriuretic peptide (ANP) levels in the children. Serum ACTH and Cor were detected by a chemiluminescence method. The detection instrument was a 180SE automatic chemiluminescence instrument manufactured by Bayer. The kit was purchased from Roche Diagnostic Products (Shanghai) Co., Ltd., and the procedures strictly followed the instructions. Serum ANP was detected by a double-antibody sandwich method. The kit was purchased from BioTek Co., Ltd. The detection instrument was a BioTek800TS multifunctional microplate reader. The inspection step was carried out strictly in accordance with the instructions. The absorbance OD value of each well was measured at a wavelength of 450 nm by the microplate reader.
(4) The self-rating anxiety scale (SAS) (12) and the self-rating depression scale (SDS) (13) were used to assess the psychological state before and after nursing, which included 20 items, with a total score of 80 points. The higher the total score is, the worse the psychological state of the children is.
(5) The Pittsburgh Sleep Index Scale (PSQI) (14) was used to evaluate the sleep quality of children in the two groups before and after nursing. The PSQI included seven items of sleep quality, sleep latency, sleep time, sleep efficiency, sleep disorders, hypnotic drugs, and daytime dysfunction, with a total score of 21 points. The higher the score is, the worse the sleep quality is. All scales were evaluated by specialist nurses.
(6) The average daily crying time, average hospitalization time, and postoperative off-bed time were compared between the two groups.

### Statistical Methods

SPSS22.0 software was used for processing. The continuous variable data of experimental data were expressed as mean ± standard deviation $\left(\bar{x}\pm s\right)$ and adopted a *t*-test. The classified variable data and descriptive analysis were expressed as % and adopted a *χ^2^* test. *p *< 0.05 indicated a significant difference.

## Results

Finally, based on inclusion and exclusion criteria, 150 children with nephroblastoma who underwent surgical treatment were included in this study.

**Figure 1:**
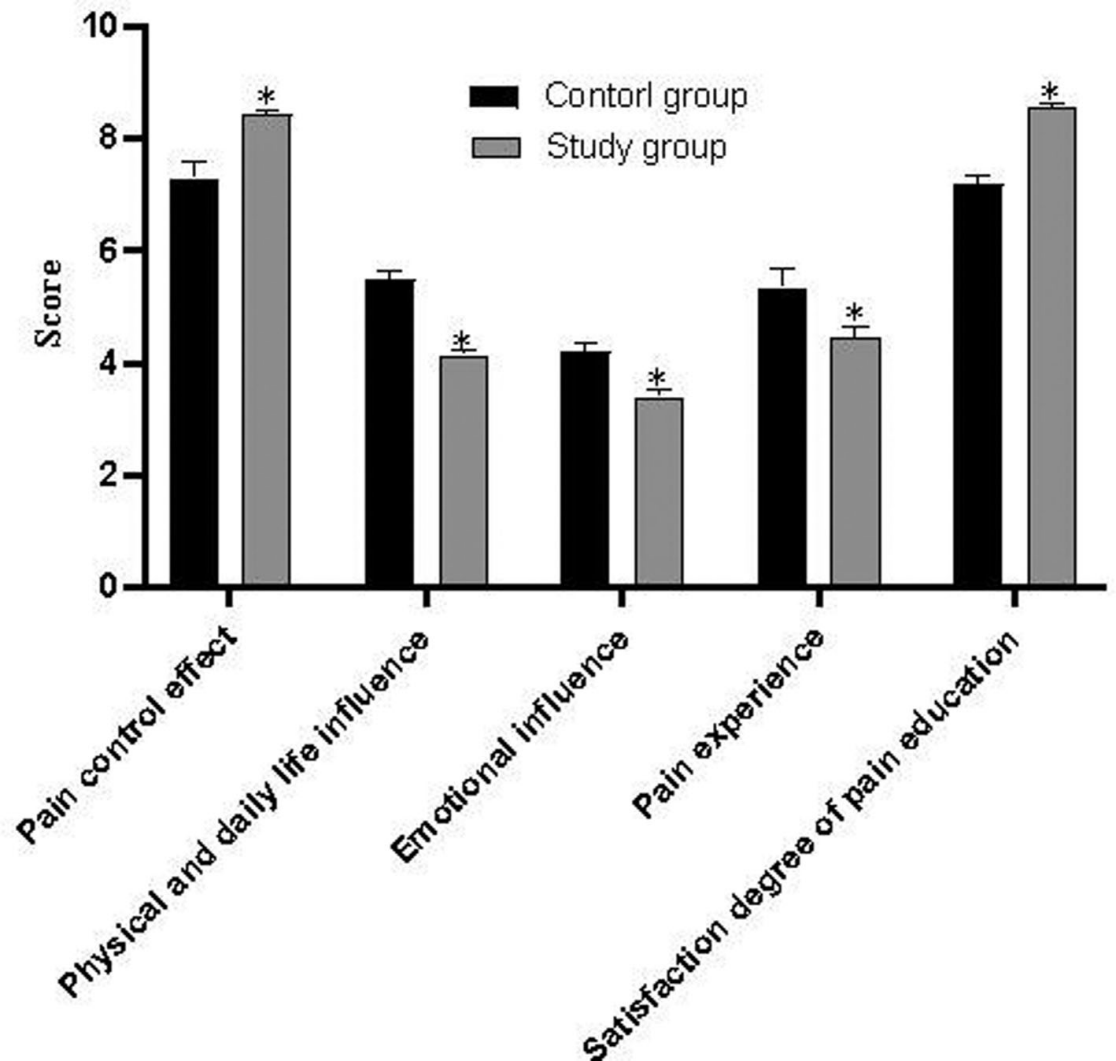
Comparison of HPOI scores between the two groups.

As shown in Figure 1, on the third day after surgery, the scores of pain control effect and satisfaction degree of pain education in the study group were higher than those in the control group, and the physical and daily life influence, emotion influence, and pain experienced in the study group were lower than those in the control group. The differences were statistically significant (*t* values were 6.662, 14.570, 21.582, 14.302, and 11.786, *p *< 0.001).

As shown in Table 1, there was no significant difference in C-SUPPH and ESCA scores between the two groups before nursing (*p *> 0.05). After nursing, the C-SUPPH and ESCA scores of the two groups were higher than those before nursing, and the C-SUPPH and ESCA scores of the study group were higher than those of the control group (*p *< 0.05).

**Table 1:** Comparison of C-SUPPH and ESCA scores between two groups of children before and after nursing ($\bar{x}\pm s$, score).

Group	*n*	C-SUPPH		ESCA	
		Before nursing	After nursing	Before nursing	After nursing
Study group	75	73.96 ± 12.59	94.51 ± 6.20*	91.98 ± 15.27	107.98 ± 10.63*
Control group	75	74.17 ± 12.47	89.59 ± 7.34*	92.66 ± 15.14	104.21 ± 10.35*
*t*	–	0.103	4.435	0.274	2.201
*p*	–	0.918	<0.001	0.785	<0.001

Note: compared with the same group before care, *p < 0.05.

As shown in Table 2, before nursing, the levels of ACTH, Cor, and ANP between the two groups were not statistically significant (*p* > 0.05). The levels of ACTH, Cor, and ANP in the two groups were lower than those before nursing and 3 d and 7 d after nursing, and the index levels after 7 d of nursing were lower than those after 3 d of nursing. After nursing, the levels of ACTH, Cor, and ANP in the study group at each time point were lower than those in the control group (*p *< 0.05).

**Table 2:** Comparison of ACTH, Cor, and ANP levels between two groups of children before and after nursing $\left(\bar{x}\pm s\right)$.

Group	*n*	ACTH(pmol/L)			Cor (nmol/L)			ANP(nmol/L)		
		Before nursing	After nursing for 3 days	After nursing for 7 days	Before nursing	After nursing for 3 days	After nursing for 7 days	Before nursing	After nursing for 3 days	After nursing for 7 days
Study group	75	30.16 ± 5.01	20.94 ± 4.86*	13.20 ± 2.51*,**	549.38 ± 24.18	430.28 ± 31.59*	408.29 ± 25.64*,**	0.75 ± 0.21	0.56 ± 0.19*	0.38 ± 0.11*,**
Control group	75	30.74 ± 5.46	26.25 ± 4.97*	16.39 ± 3.17*,**	538.63 ± 24.78	459.69 ± 30.74*	426.98 ± 27.36*,**	0.76 ± 0.23	0.64 ± 0.21*	0.44 ± 0.18*,**
*t*	–	0.678	6.616	6.832	2.689	5.778	4.317	0.278	2.446	2.463
*p*	–	0.499	<0.001	<0.001	0.008	<0.001	<0.001	0.781	0.016	0.015

Note: compared with the same group before care, *p < 0.05. Compared with the same group after nursing for 3 days, **p < 0.05.

As shown in Table 3, there was no significant difference in SAS and SDS scores between the two groups before nursing (*p* > 0.05). After nursing, the SAS and SDS scores of both groups were lower than those before nursing, and the SAS and SDS scores of the study group were lower than those of the control group (*p *< 0.05).

**Table 3:** Comparison of SAS and SDS scores between two groups of children before and after nursing ($\bar{x}\pm s$, score).

Group	*n*	SAS		SDS	
		Before nursing	After nursing	Before nursing	After nursing
Study group	75	56.39 ± 8.12	40.20 ± 6.24*	51.96 ± 7.33	39.37 ± 3.67*
Control group	75	57.84 ± 8.33	46.33 ± 7.25*	50.85 ± 7.49	43.23 ± 4.02*
*t*	–	1.080	5.550	0.917	6.141
*p*	–	0.282	<0.001	0.361	<0.001

Note: compared with the same group before care, *p < 0.05.

As shown in Table 4, there was no significant difference in PSQI scores between the two groups before nursing (*p *> 0.05). After nursing, the PSQI scores of the two groups were lower than those before nursing, and the PSQI scores of the study were lower than those of the control group (*p *< 0.05).

**Table 4:** Comparison of PSQI scores between two groups of children before and after nursing ($\bar{x}\pm s$, score).

Group	*n*	Before nursing	After nursing
Study group	75	15.29 ± 2.16	10.93 ± 1.38*
Control group	75	15.87 ± 2.37	12.45 ± 2.15*
*t*	–	1.566	5.153
*P*	–	0.119	<0.001

Note: compared with the same group before care, *p < 0.05.

As shown in Table 5, the average daily crying time, the average hospitalization time, and postoperative off-bed time in the study group were shorter than those in the control group (*p *< 0.05).

**Table 5:** Comparison of average daily crying time, average hospitalization time, and postoperative off-bed time between two groups of children $\left(\bar{x}\pm s\right)$.

Group	*n*	Average daily crying time (min/d)	Average hospitalization time (d)	Postoperative off-bed time (d)
Study group	75	35.95 ± 6.07	11.39 ± 2.52	3.01 ± 1.06
Control group	75	40.21 ± 5.82	13.48 ± 2.14	3.49 ± 1.25
*t*	–	4.387	5.475	2.536
*p*	–	<0.001	<0.001	0.012

## Discussion

Nephroblastoma is currently the most important malignant solid tumor threatening the life safety of children, and surgery is the most important treatment in clinical practice. Pain is one of the most important complications in these types of patients after surgery. Moreover, children have poor self-control ability and are weak, and their tolerance to pain is much lower than that of adults (15). However, studies have shown that effective pain care for children undergoing nephroblastoma surgery is very important to promote postoperative rehabilitation; so, seeking an effective nursing plan has become the focus of clinical attention.

Severe pain will aggravate adverse psychological stress and promote adverse neuroendocrine and metabolic reactions in the body. Also, malignant stress is often not conducive to the recovery of children’s condition (16). Some studies have pointed out that self-care ability can improve the clinical outcome and psychological state of patients undergoing surgery to a certain extent and maintain and promote health status.

In this study, the results showed that after nursing, the C-SUPPH and ESCA scores of the study group were higher than those of the control group, while the stress response index level, psychological state score, and PSQI score were lower than those of the control group (*p *< 0.05). This indicates that the implementation of the nursing plan based on Orem’s self-care theory can not only physiologically alleviate the pain of children and alleviate the stress of surgery but also effectively alleviate the anxiety and depression of children after surgery and improve their sleep quality. Self-care theory is a comprehensive nursing model proposed by Dorothea E. Orem, a famous contemporary American nursing theorist (17). The main purpose of self-care theory is to help patients improve their consciousness and ability of self-care on the basis of all-round and comprehensive nursing and also play a role in improving their psychological status (18). In our study, during the period of nursing care, through constantly stimulating the subjective initiative of children, their psychological status was changed so that they could actively participate in the treatment and gradually take up the responsibility of care for themselves. Unlike control group patients who received the traditional care model, under the support of personalized care plan, patients in the research group were strictly observed for their self-care consciousness, psychological state, and self-care ability, so the nursing effect was obviously superior to that in the control group.

Assessment of postoperative active pain in children is also an effective means to relieve pain in children (19). At present, most hospitals in many countries around the world have established assessment programs for postoperative active pain, but little attention has been paid to the effect of postoperative pain on functional activities in China (20). Some scholars pointed out that the application of active pain assessment in clinical practice can be used as the basis for the evaluation of analgesic efficacy and treatment of active pain, which can effectively control the pain in patients and improve the quality of postoperative pain management.

It has been pointed out that subjective evaluation tools can directly reflect the subjective pain feelings of patients and provide a certain reference for formulating appropriate intervention measures. NRS is a commonly used assessment tool for clinical evaluation of patients’ subjective pain in the past (21). However, the defect of this scale lies in that it can only reflect patients’ subjective pain perception, cannot objectively reflect patients’ ability to complete a functional activity, and cannot assess patients’ actual activities, thus affecting the treatment of active pain in patients after surgery. FAS is an objective application evaluation tool with medical staff as the main body. Medical staff observe the completion of functional activities of patients and make the corresponding evaluation of activity level, which can make up for the deficiencies of NRS to a certain extent (22).

The results of this study show that the control group only used the NRS score to understand the functional activities of the patients, while the study group used the NRS score for the subjective feelings of the patients and the four-level FAS score objectively evaluated by medical staff to evaluate the pain severity of the patients, which provided a valuable reference index for the treatment of active pain and thus promoted the improvement of the quality of postoperative pain management. The evaluation results based on the HPOI scale showed that the scores of pain control effect and pain education satisfaction in the study group were higher than those in the control group, and the scores of body and daily life influence, emotion influence, and pain experienced in the study group were lower than those in the control group with statistically significant differences (*p *< 0.05), which confirmed the application value of NRS combined with FAS scale activity evaluation in pain care for children with nephroblastoma after operation. In addition, the daily crying time, average hospital stay, and the first time to get out of bed in the study group were shorter than those in the control group (*p *< 0.05). It also indicates that postoperative self-care for children with nephroblastoma and effective pain assessment with appropriate assessment tools have significant advantages in promoting postoperative recovery and shortening treatment time.

## Conclusion

In summary, Orem’s self-care theory combined with active pain assessment can reduce pain in children undergoing nephroblastoma surgery, improve their stress response and psychological state, and improve their sleep quality, which is conducive to postoperative recovery and worthy of promotion.

## Data Availability

The original contributions presented in the study are included in the article/Supplementary Material; further inquiries can be directed to the corresponding author/s.
